# Global Climate Change and Children’s Health: Threats and Strategies for Prevention

**DOI:** 10.1289/ehp.1002233

**Published:** 2010-10-14

**Authors:** Perry E. Sheffield, Philip J. Landrigan

**Affiliations:** 1 Department of Preventive Medicine and Pediatrics and; 2 Children’s Environmental Health Center, Department of Preventive Medicine, Mount Sinai School of Medicine, New York, New York, USA

**Keywords:** environmental justice, global burden of disease, global warming, pediatric environmental health, vulnerable populations, weather

## Abstract

**Background:**

Global climate change will have multiple effects on human health. Vulnerable populations—children, the elderly, and the poor—will be disproportionately affected.

**Objective:**

We reviewed projected impacts of climate change on children’s health, the pathways involved in these effects, and prevention strategies.

**Data sources:**

We assessed primary studies, review articles, and organizational reports.

**Data synthesis:**

Climate change is increasing the global burden of disease and in the year 2000 was responsible for > 150,000 deaths worldwide. Of this disease burden, 88% fell upon children. Documented health effects include changing ranges of vector-borne diseases such as malaria and dengue; increased diarrheal and respiratory disease; increased morbidity and mortality from extreme weather; changed exposures to toxic chemicals; worsened poverty; food and physical insecurity; and threats to human habitation. Heat-related health effects for which research is emerging include diminished school performance, increased rates of pregnancy complications, and renal effects. Stark variation in these outcomes is evident by geographic region and socioeconomic status, and these impacts will exacerbate health disparities. Prevention strategies to reduce health impacts of climate change include reduction of greenhouse gas emissions and adaptation through multiple public health interventions.

**Conclusions:**

Further quantification of the effects of climate change on children’s health is needed globally and also at regional and local levels through enhanced monitoring of children’s environmental health and by tracking selected indicators. Climate change preparedness strategies need to be incorporated into public health programs.

Global climate change is anticipated to increase the average global temperature and the frequency of extreme weather events. Regional projections, however, show substantial variation in amount and timing of precipitation, increasing in some places and decreasing in others, and they indicate an overall increase in variability of weather patterns (Bernstein et al. 2007). Sea level rise, another consequence of climate change, will lead to flooding especially in island nations and low-lying delta regions (Bindoff et al. 2007). These diverse changes will have varied and numerous health impacts. Few of the many publications describing health impacts of climate change focus on effects among children or other vulnerable subpopulations [Balbus and Malina 2009; World Health Organization (WHO) 2009b]. Table 1 summarizes the outcomes most commonly examined (Akachi et al. 2009; Bunyavanich et al. 2003; Canadian Institute of Child Health 2001; Ebi and Paulson 2007; Shea et al 2007; United Nations Children’s Fund 2007; U.S. Environmental Protection Agency 2009).

Using a children’s health framework (Daston et al. 2004; Landrigan et al. 2004), in the present review we summarize children’s vulnerability to climate-related environmental hazards, emphasizing that the disproportionate impacts will exacerbate existing issues of environmental justice (Council on Community Pediatrics and Committee on Native American Child Health 2010; Landrigan et al. 2010). We focus on the health impacts of major exposures associated with climate change, namely, increased temperatures, increasing frequency and severity of weather extremes, and sea level rise. We do not focus on impacts from adaptation and mitigation decisions that also have the potential to affect children’s health. Notably, health impacts from adaptation and mitigation could be positive, especially if they reduce other negative environmental exposures. We then explore the current state of evaluation of pediatric disease burden associated with climate change using the epidemiologic framework proposed by McMichael (2001a) (Table 1). Last, we review prevention strategies to reduce impacts of global climate change on children’s health.

## Children’s Vulnerability to Environmental Exposures: Science and Social Justice

The exposures that influence children’s health begin before conception—reflecting parents’ diets and other environmental exposures—and continue through pregnancy, childhood, and adolescence. Table 2 presents some examples of climate-sensitive exposures to children at each life stage. Several factors may alter children’s environmental exposures relative to adults and increase children’s susceptibility to the effects of such exposures or stresses. These include the following six elements with climate-sensitive examples.

### Differences in physiology and baseline metabolism

Children have less effective heat adaptation capacity than do adults (Committee on Sports Medicine and Fitness 2000).

### Early rapid development creates windows of vulnerability *in utero* (Selevan et al. 2005) and in early childhood

Exposures during these windows can cause devastating damage that has no counterpart in adult life (Berkman et al. 2002). When children contract *Plasmodium falciparum* malaria from mosquitoes, a vector exquisitely sensitive to changes in temperature and precipitation, they have a higher complication rate (severe anemia, cerebral malaria, and long-term neurologic sequelae) and a higher mortality rate relative to older populations, presumably because they have less acquired functional immune response (Patz and Reisen 2001; Snow et al. 1999). Similarly, prenatal or childhood exposure to specific toxins, toxicants, infectious agents, or conditions such as undernutrition can produce disease and dysfunction that lasts through childhood and in some cases first manifests only in adulthood (Crimmins and Finch 2006; Hales 1997; Victora et al. 2008).

### Higher exposures per unit body weight

Because they breathe more air, drink more water, and eat more food per unit of body weight, children experience greater proportionate exposure than do adults to, for example, ground-level ozone on high-air-pollution days or pesticides in drinking water (Kim et al. 2004; Landrigan and Garg 2005).

### Different diet and behaviors

Children consume a larger relative proportion of fruits and vegetables and spend a larger proportion of time outdoors than do adults, increasing climate-sensitive exposures such as pesticide residues on food and outdoor insect vectors (Pronczuk 2005).

### More future years of life

Children are expected to be alive longer than adults, exposing them to newly developing or worsening environmental hazards in the future. Additionally, many diseases have a long latency period, sometimes requiring decades to develop (Landrigan and Garg 2005).

### Dependence on caregivers

Children’s health can be affected not only by health problems or other impairment of caregivers but also by their reliance on adults as political proxies making decisions that have long-term societal impacts. Thus, children’s vulnerability is intergenerational as well as biologic (Ebi and Paulson 2007; United Nations Population Fund 2009).

#### Current global burden of climate-related disease on children

Approximately one in five deaths around the world each year occurs in a child < 5 years of age (WHO 2008). Lower respiratory tract infections, diarrhea, and malaria are responsible for > 50% of childhood deaths (Prüss-Üstün and Corvalan 2007). All three of these disease categories could worsen with climate change. Diarrheal disease is primarily attributable to environmental factors, specifically contaminated food and drinking water (WHO 2008), and is affected by changing temperature and precipitation events (Campbell-Lendrum and Woodruff 2006). Thirty-five percent of excess child mortality is secondary to malnutrition (Black et al. 2008), a risk factor also expected to worsen with climate change because of increasing food insecurity (McMichael 2001b). Micronutrient deficiencies, common with malnutrition, can exacerbate infectious disease morbidity (Bhutta et al. 2008).

**Figure 1:**
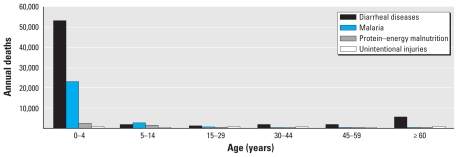
Deaths attributable to global climate change: 2004 annual data in total numbers divided by age categories (adapted from WHO 2008).

**Figure 2:**
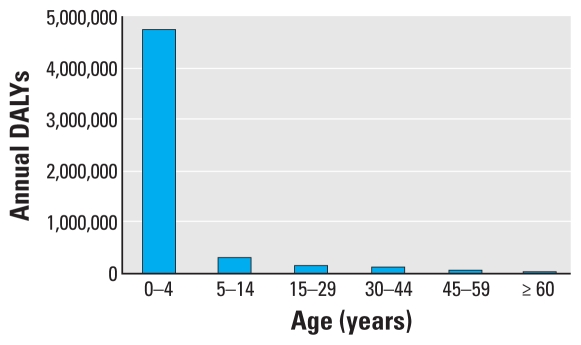
DALYs attributable to global climate change: annual data in total numbers divided by age categories (aadapted from WHO 2002a).

The WHO estimates global burden of disease using the disability-adjusted life year (DALY) metric that includes morbidity as well as mortality and provides a composite picture of health impacts caused by diverse risk factors (Kovats et al. 2005). According to WHO estimates using the DALY metric, > 88% of the existing burden of disease due to climate change occurs in children < 5 years of age in both developed and developing countries (Zhang et al. 2007). This estimate is markedly higher than the pediatric proportion of the total burden of disease, which for children < 5 years of age is 5% in high-income countries and 31% in low- and medium-income countries (WHO 2008). Zhang et al. (2007) argue that DALYs are important when assessing climate change impacts to establish priorities and to evaluate the efficiency of environmental policies. Figures 1 and 2 show, respectively, the annual deaths attributable to climate change for four significant disease categories and the climate-related DALYs lost among different age groups. Children suffer a much greater burden of climate-related disease than do adults (WHO 2002a, 2008). Furthermore, the impacts of climate change on children are not evenly distributed globally, but instead occur in parts of the world already experiencing a higher relative disease burden, namely, in low-income countries (Haines et al. 2006).

Globally, for all ages, the burden of disease attributable to climate change in the year 2000 was > 150,000 deaths (0.3% of global deaths) and 5.5 million lost DALYs (0.4% of global burden) (McMichael et al. 2004; WHO 2002a). These estimates from 2000, which are the most current available, are likely conservative because they include only five health outcomes: direct temperature effects, diarrhea, malnutrition, flood-related injury, and malaria.

#### Changing climate-sensitive issues and diseases affecting children

McMichael (2001a) set out an epidemiologic framework with three primary challenges: *a*) establishing response functions for climate-sensitive diseases using existing data sets, *b*) conducting targeted surveillance to detect changes in current climate-sensitive disease outcomes, and *c*) projecting climate-sensitive health burdens. Above we reviewed efforts to quantify the existing global burden of climate-sensitive disease among children, which builds on the first two of McMichael’s tasks. We now focus on some specific climate-sensitive health risks that also build on baseline epidemiology but further detail the mechanisms underlying these child-specific risk factors.

### Changing rates of infectious disease

Global climate change will likely increase the spread of some infectious diseases categorized as vector-borne, food-borne, and water-borne diseases. Among the vector-borne illnesses, the climate health impact literature focuses primarily on malaria, dengue fever, and tick-borne diseases such as Lyme disease. Complications from a number of such diseases, particularly malaria, are higher in children. Regarding diarrheal diseases, a number of studies have shown links between temperature or rainfall events and generic acute gastrointestinal illness or specific food- or water-borne illnesses (Drayna et al. 2010; Fleury et al. 2006; Singh et al. 2001; Thomas et al. 2006; Zhang et al. 2008). One study looked specifically at pediatric diarrheal disease incidence and temperature. During an El Niño year when temperatures were up to 5°C above normal in Lima, Peru, diarrheal hospitalization rate among children increased to 200% of the previous rate (Checkley et al. 2000). Another potential health threat is rapid human migration, which increases the chances of large-scale exposure of immunologically naive populations to infectious diseases (Patz and Reisen 2001).

### Malnutrition

The 2002 World Health Report states that childhood malnutrition is the most widespread and pervasive primary risk factor for the major diseases of children (WHO 2002b). Climate change may worsen malnutrition by directly affecting agricultural yields and worsening growing conditions in areas already experiencing food insecurity (Parry et al. 2005). Changes in seasonality in regions with distinct periods of rainfall, heavy rainfall events, and droughts can all negatively affect children’s nutritional status (Choudhury and Bhuiya 1993; Maleta et al. 2003; Singh et al. 2006). In addition, there are important interactions between infection and malnutrition, resulting in increased risk of complications from infection if a child is malnourished and vice versa (Mihrshahi et al. 2007; Shell-Duncan 1995). Nutritional impacts of climate change are difficult to quantify because they result from a composite of macrosocial factors. However, climate change impacts on malnutrition, as well as on infectious disease, will likely continue to represent the largest proportion of the burden of disease on childhood morbidity and mortality attributable to climatic change.

### Allergic and nonallergic disease from air pollution and allergens

Numerous air pollutants with well-established pediatric respiratory effects (Silverman and Ito 2010; Trasande and Thurston 2005) will potentially change as climate changes. Ozone is expected to increase in some regions, and other pollutants, such as nitrogen oxides, particulate matter, and sulfur oxides, will also potentially change because warming temperatures can affect chemical reaction rates and pollutant transport mechanisms (Ebi and McGregor 2008; Kinney 2008). Wildfires, also a climate-sensitive exposure, can generate significant particulate matter and have been documented to increase risk of respiratory effects and eye irritation among children in affected areas (Kunzli et al. 2006).

Weed pollen, shown to increase in climate change simulation studies, and grass pollen have been associated with children’s asthma exacerbations, emergency department visits, and hospitalizations (Héguy et al. 2008; Schmier and Ebi 2009; Ziska et al. 2008). The last several decades have shown an increasing global trend in the incidence of asthma and potentially other allergic diseases, and some hypothesize that increased aeroallergen exposure from climate change might be partially responsible (Beggs and Bambrick 2005; Beggs and Walczyk 2008). However, more deliberative scientific bodies have not made this same assertion.

### Extreme storm events, increased extreme heat, and sea level rise

Health impacts on children from projected increases in frequency and severity of extreme storm events and sea level rise that result from global warming include acute injuries, chronic mental illness, food insecurity issues, food and water contamination, and potential wide-scale population displacement. All of these topics have been examined extensively in previous review articles (Akachi et al. 2009; Bunyavanich et al. 2003; Canadian Institute of Child Health 2001; Ebi and Paulson 2007; Shea et al. 2007; United Nations Children’s Fund 2007; U.S. Environmental Protection Agency 2009). One additional study demonstrated that, during the 2006 heat wave in California, 0- to 4-year-old children had increased emergency department visits for electrolyte imbalances compared with periods without heat waves, an increase that was essentially the same as for older children and adults [relative risk (RR), 1.19 vs. 1.18] (Knowlton et al. 2009). Although the risk of heat-related deaths among children in the United States is lower than the risk among the elderly, it is still higher than the general population (Moore et al. 2002).

In the 1990s, disasters affected 66.5 million children around the world (Penrose and Takaki 2006). Estimates for the future range as high as 175 million children per year (Save the Children UK 2007). Extreme storms such Hurricane Katrina in the United States highlight the specific challenges posed by the pediatric population even in countries with significant public health capacity (Johnston and Redlener 2006). An additional impact is that of climate-forced migration leading to climate-change refugees. This forced migration or population displacement is expected to affect health, economic development, and political instability, perpetuating cycles of poverty and civil unrest that already contribute substantially to the global burden of human disease (Confalonieri et al. 2007) and impede environmental justice.

There are also potentially subclinical effects from extreme heat events. The impacts include decreased functioning and diminished productivity (Kjellstrom et al. 2009). Although heat can potentially affect cognitive performance, we found limited studies, such as Dapi et al. (2010), quantifying the cognitive effects of increased heat on student learning and performance in schools that are not climate controlled.

### Renal effects

In one London-based study, children 0–4 years of age showed hospitalization rates above baseline not only for respiratory but also for renal disorders in relation to increasing ambient summer temperatures (Kovats et al. 2004). In addition, Mandeville and Nelson (2009) report a positive correlation between increased ambient temperature and urolithiasis. Heat-related dehydration was the suspected underlying cause. Dehydrated children are vulnerable to renal effects because dehydration, especially if associated with increased perspiration, triggers lower urine volume and higher supersaturation of stone-forming salts (Fakheri and Goldfarb 2009). One study projected an increase in all-age urolithiasis incidence based on future temperature changes (Brikowski et al. 2008). We found no articles quantifying the climate-related burden of disease on children from urolithiasis or addressing other kidney disease.

### Pregnancy and prenatal complications

Study of the potential influences on pregnancy and prenatal complications of direct temperature effects related to climate change is an area of emerging research (Confalonieri et al. 2007). There is some evidence that exposure to extreme heat during pregnancy is related to lower birth weight, and this effect appears to be most important when exposure occurs in the second and third trimesters on U.S. births (Deschênes et al. 2009). The clinical significance of the decrease in birth weight observed in these studies is not clear. Ambient temperature during gestation has also been observed to affect sex ratio at birth and longevity of males in a Northern European population (Catalano et al. 2008), with mechanisms that are not clear. Also, there is some evidence of associations between climatic variables, such as increased humidity, and preeclampsia and eclampsia (prenatal complications), which present health risks not only for the unborn child but also for the mother (Subramaniam 2007). Finally, particulate air pollution is associated with preterm births, low birth weight, and infant mortality (Kim et al. 2004). As noted above, climate change could alter concentrations of particulate matter as a result of increased wildfires or changes in pollutant transport mechanisms and thus influence pregnancy outcomes.

### Livelihoods, food security, and human security

Poorer households have been observed to be more vulnerable to climate change–related events such as floods (Brouwer et al. 2007), and children, especially those in poverty, would be likely to be more affected by such events (UNICEF Innocenti Research Centre 2008). A disruption of livelihood can affect not only susceptibility to disease but also adaptive capacity (Hahn et al. 2009). A particularly difficult area to quantify is the potential effect of climate change on war or political instability resulting from mass migrations; local security changes from increased environmental or psychosocial stress; and social disruption due to extreme events and livelihood disruption (Barnett 2003; Sondorp and Patel 2003). One study shows an association of increased temperatures and armed conflict in Africa (Burke et al. 2009). Social disruption often has disproportionate impacts on girls, including increased health impacts and workloads and decreased educational access (UNICEF Innocenti Research Centre 2008).

### Toxic exposures

Children warrant special consideration when quantifying the impact of potential toxic exposures because of their potential increased susceptibility to negative health effects during their rapid growth and development and greater exposures per body weight. Changes in temperature, humidity, and the hydrologic cycle will affect patterns of exposure to chemicals used in food production and other pest control (Confalonieri et al. 2007). An investigation into agricultural pesticide use and temperature and precipitation changes showed an overall increase in cost expected from likely climatic changes (Chen and McCarl 2001). Although most current pesticides are not as persistent or bioaccumulative as those used in the past, their water solubility increases the risk of water contamination, particularly after extreme precipitation events (Donald et al. 2005; Dwight et al. 2002). The extent of human exposure and health effects under future climate change will depend on adoption of less-toxic practices that account for changes in such factors as temperature and precipitation (Boxall et al. 2009).

Chemicals known as persistent organic pollutants (POPs) last for decades without biodegrading. Global surface temperatures, wind patterns, animal migratory patterns, and global ice volume play a role in the distribution of these chemicals (Carrie et al. 2010). Climate change is expected to result in a changing global distribution of heavy metals (e.g., mercury) and POPs as well as altered biotransformation (Booth and Zeller 2005; Noyes et al. 2009). Some climate factors will be expected to speed the transformation process, thereby decreasing the global load of these chemicals, but the altered distribution could mean that some regions of the world experience increased deposition. POPs and other global pollutants such as mercury and lead are already known to have both acute and chronic effects on children. In addition to neurodevelopmental disorders, the health effects from these varied chemicals include endocrine disruption and carcinogenicity (Diamanti-Kandarakis et al. 2009; Eskenazi et al. 2009; Makalinao and Woolf 2005).

Another potential climate-related change in toxic exposure could result from increased contamination in grain and legume crops by mycotoxin-producing fungi, with both acute and chronic health effects in livestock and humans. Aflatoxin (one type of mycotoxin) is produced by *Aspergillus* species. Risk of contamination depends on many factors such as regional climate, preseason precipitation, minimum and maximum daily temperature, and daily net evaporation (Ono et al. 1999; Strosnider et al. 2006). More than four billion people in developing countries are at risk of chronic exposure to aflatoxin (Williams et al. 2004), which increases the risk of hepatocellular carcinoma, impaired growth, and immune suppression (with unknown clinical significance). During acute aflatoxicosis outbreaks manifesting as hepatitis and jaundice, children have a higher reported mortality rate (Strosnider et al. 2006; Williams et al. 2004). Other health effects of mycotoxins include other cancers, ergotism, and higher rates of birth defects (Etzel 2002). Research and policy change will be necessary to avoid health effects of changing exposure to natural and man-made agricultural contamination (Boxall et al. 2009).

#### Future projections of children’s health impacts from climate change

Estimating the future burden of climate-related disease impacts on children is complex. Future projections should take into account not only local climate-related effects but also other significant environmental changes such as stratospheric ozone depletion, accelerating loss of biodiversity, and alterations in elemental cycles such as the nitrogen cycle (McMichael 2001b). Although in some geographic areas and for some diseases the effects of climate change may be beneficial, overall global climate change is projected to increase the global burden of disease (Hitz and Smith 2004).

McMichael et al. (2004) estimated RRs for exposures to thermal extremes and weather disasters (deaths and injuries associated with floods), distribution and incidence of malaria, incidence of diarrhea, and malnutrition (via effects on yields of agricultural crops) in 2030 for each of the WHO subregions. Overall, extreme weather accounted for the largest proportional RR change (RRs for inland floods up to 18.5) but diarrheal disease (RRs up to 1.1) and malaria (RRs up to 1.83) accounted for the larger burden of disease using unmitigated emissions scenarios. Malnutrition RR projections varied highly by world region. These are not age-stratified assessments. Despite projections of increased yields in some more temperate regions of the world, sub-Saharan Africa is projected to have increased food insecurity. By the 2080s, overall yield changes are projected to result in an additional 70 million hungry people globally, 40% more than expected (McMichael 2001b). These impacts would perpetuate a disproportionate burden of malnutrition on children.

Many countries with the highest rates of childhood disease and death are undergoing demographic transitions toward greater industrial development. However, according to the 2002 World Health Report (WHO 2002b), even after accounting for such transition, underweight is still expected to be in the top five causes of global DALYs in the year 2020, thus reflecting an ongoing vulnerability to climatic-induced changes in food security in many poor countries. The climate health research community will be producing updated and age-stratified projections in the near future. Such projections will help inform public health interventions.

### Prevention through adaptation, resilience, and mitigation

Prevention strategies in relation to climate change have largely focused on reduction or mitigation of greenhouse gas (GHG) levels in the global system. However, many GHGs in the atmosphere and dissolved in the ocean from past and ongoing human activity are long-lived and will drive climate change for years to come, a process termed “built-in” climate change (Solomon et al. 2009). Their continuing presence argues for the need to develop evidence-based adaptation strategies that proceed in parallel with efforts to prevent GHG accumulation.

The concept of prevention in public health is multitiered. Table 3 portrays the levels of prevention in relation to potential health effects from climate change, with child-protective examples at each level. Primary, secondary, and tertiary prevention all have a role in adaptation, or preparedness, and each can contribute to the resilience of individuals, communities, and nations. Resilience is defined as “the ability of a social or ecologic system to absorb disturbances while retaining the same basic structure and ways of functioning, the capacity for self-organization, and the capacity to adapt to stress and change” (Baede et al. 2007). Although public health efforts aimed at any of these levels of prevention typically benefit children, some prevention resources are best spent on specifically targeting children or their parents.

### Children’s environmental health indicators

Previous articles on the impacts of climate change on health, and on children’s health in particular, have called for additional research to improve understanding of the relationships between climatic factors and health, quantification of the current impacts, and projections of future impacts. Such research, including surveillance and program evaluation, is essential to inform the prioritization of activities related to climate change adaptation in public health. Frameworks for prevention include incorporation of climate change actions into the 10 essential functions of public health (Frumkin et al. 2008) and the WHO’s efforts to develop internationally comparable children’s environmental health indicators (CEHIs) (WHO 2009a). CEHIs—subdivided into categories of context, exposures, health outcomes, and actions—have emerged from several international agreements as a proposed tool for tracking the state of children’s environmental health (WHO 2009a). Table 4 gives some examples of CEHIs linked to climate change impacts. Specifically, there is a need for quality and continuous environmental health indicator data that are disaggregated by age group and include children (Akachi et al. 2009). If standardized CEHIs are collected regionally, comparisons among areas have more validity and targeted interventions can be tailored to be regionally specific. The goals of such efforts involve getting a better handle on the current burden of disease, following trends, identifying hot spots, and consequently enabling better prioritization and capacity building. The argument for children’s increased vulnerability and their disproportionate burden of disease presented in this article underscores the importance of child-specific information to respond to their sensitivities and disproportionate exposures.

### Existing strategies

A reasonable public health strategy addresses present-day health problems while building adaptive capacity to respond to worsening future impacts from climate change. Although climate change is already creating a substantial global burden of disease, the current DALYs due to unsafe water, lack of sanitation and hygiene, urban air pollution, indoor smoke from solid fuels, and lead exposure dwarf the present-day effects of climate change on health (WHO 2002a).

Numerous programs exist into which children’s environmental health platforms, with specific attention to climate change adaptation, could be incorporated (St Louis and Hess 2008). Integration of climate change adaptation within global health strategy could mean both better sustainability of the existing programs as the climate becomes increasingly unpredictable and better inclusion of these climate change efforts in the near-term global health programs by coupling with programs that already have funding. In addition, climate change preparedness is key to the long-term sustainability of most of the United Nations’ eight Millennium Development Goals, with a target year of 2015: eradicating extreme poverty and hunger (goal 1), promoting gender equality (goal 3), reducing child mortality (goal 4), improving maternal health (goal 5), combating HIV/AIDS, malaria, and other diseases (goal 6), and ensuring environmental sustainability (goal 7) (United Nations Development Programme 2009). Climate change is expected to act primarily as an effect modifier in exacerbating existing health disparities, and thus its inclusion in planning is essential for the success of global health efforts (McMichael and Butler 2004; Witherspoon 2009).

Another area that integrates well with pediatric prevention efforts around climate change is the global demand from women for increased access to birth control (Costello et al. 2009). Although a reduced birth rate does not necessarily equate with reduced GHG emissions, family planning via provision of contraception, female literacy, and education, as well as property rights for women, will reduce poverty and aid sustainable development (United Nations Development Programme 2009). Increased access to desired birth control would result in a reduction of potentially 200 million unintended pregnancies per year. Additionally, spacing of pregnancies could protect unborn children, because intervals of 15 and 75 months between pregnancies result in lower rates of fetal loss (DaVanzo et al. 2007; United Nations Population Fund 2009). Health benefits for young children are also possible, because some studies suggest that in certain populations short birth intervals have a negative effect on children’s nutritional status (Dewey and Cohen 2007). In addition, there is evidence for a window of ideal spacing of children for the mother’s health. Maternal morbidity and mortality decrease if the interpregnancy interval is > 6 months but < 5 years (Conde-Agudelo and Belizán 2000). Interventions that promote family planning can thus potentially reduce children’s exposure and susceptibility to climate-sensitive impacts.

Last, children can play a role in adaptation, namely in building resilience. Children should attend school where opportunities exist for curriculum development that best prepares them for expected changes. There are international precedents for such action in the sustainable-school and health-promoting schools movements (Davis and Cooke 2007).

### New programming

New prevention projects should incorporate adaptations whose impacts have been carefully considered for unintended consequences so that they can simultaneously improve existing health disparities, build community resilience, and decrease climate-related impacts (Garg et al. 2009). Local public health efforts can make substantial differences in the morbidity and mortality from natural disasters (Keim 2008) as well as the influence of climate on vector-borne diseases. Resilience can be increased through proper preparation and engagement of local stakeholders in scenario-based preparation (Ebi and Semenza 2008), as well as through use of climate-risk expertise in the insurance industry to inform public policy (Crichton 2007). Within the United States, a community’s relationship with its location—its “sense of place”—has implications for the motivation, development, and implementation of an effective public health response to prepare for climate change (Hess et al. 2008).

An emerging tool to assess new policies, programs, and projects is the health impact assessment. In climate change work, health impact assessments are an emerging practice that strives to quantify the positive and negative health effects of an intervention (Patz et al. 2008). The cost assessment of a capacity-building project or other adaptation measure should include risks and benefits of alternative measures (e.g., increased used of chemical fertilizers and pesticides in response to decreasing crop yields or increased pest burdens) and also of inaction. The assessment should also include the potential reduction in current non-climate-related burden of disease (i.e., health co-benefits). A shift in agricultural production away from livestock and toward provision of plant-based diets is one example of a means to reduce cardiovascular disease and GHG production (Friel et al. 2009). Other studies have quantified co-benefits of improved respiratory health, reduction of missed work days, and reduction of premature deaths—all derived from improved air quality that would result from lower air concentrations of ozone and particulate matter through mitigation activities (e.g., improved cookstoves) that reduce GHG emissions (Cifuentes et al. 2001). Both *Lancet* (2009) and the National Institute of Environmental Health Sciences (2009) are putting substantial effort into exploring the public health impacts, including co-benefits, of GHG mitigation. Both mitigation and adaptation efforts coupled with existing public health strategies and also developed as new programs are essential to reduce the increasing burden of disease on children from climate change.

## Conclusion

The health impacts of global climate change are expected to be widespread, geographically variable, and profoundly influenced by preexisting social and economic disparities. Effects on children and on other vulnerable populations are already—and are projected to continue to be—disproportionately heavy. The literature on proven and plausible health impacts of global climate change now covers virtually every organ system in the human body. More thorough quantification of child-specific health impacts of global climate change is urgently needed.

The expectation that global climate change will produce wide-scale, but still inadequately quantified, increases in disease and death warrants a societal commitment that combines ongoing research with vigorous and thoughtful efforts to reverse health impacts and reduce worsening environmental injustice. Prevention efforts directed against the health effects of climate change should acknowledge the inherent vulnerabilities of children and seek to reduce both their exposures and susceptibility. Health projects that benefit adults while appropriately protecting children will have added benefits for the children by protecting their caregivers.

Specific needs include enhanced monitoring of current children’s environmental health status, better incorporation of climate change adaptation into existing programs, and new climate-sensitive disease prevention programs that have short- and long-term health co-benefits.

## Figures and Tables

**Table 1:** Child-specific, climate-sensitive health risks and effects^a^ through the lens of global climate change as a risk factor and resulting epidemiologic challenges.^b^

Child-specific, climate-sensitive health risks and effects	Climate–health response function determinants	Current preventable climate-sensitive disease burden globally	Models of future burden of disease
Infectious disease	Vector control programs, water and sanitation infrastructure, immunization status	More than 80,000 annual deaths due to malaria and diarrheal disease in children < 15 years of age; DALYs not quantified by category	No age-stratified projections exist by disease category
Food, water, toxics	Food and water availability, access, and quality	Almost 4,000 deaths from protein–energy malnutrition in children < 15 years of age; DALYs not quantified by category	
Air contaminants	Particulate matter, ground-level ozone, and other pollutant levels; air quality alerts; pollen timing and intensity	Unquantified	
Extreme weather	Early warning systems, emergency preparedness plans, baseline infrastructure	Almost 1,000 annual deaths from unintentional injury associated with extreme weather	
Population displacement	Political stability, services available to climate refugees	Unquantified	

a Adapted from Akachi et al. (2009), Bunyavanich et al. (2003), Ebi and Paulson (2007), and Shea et al. (2007).

b Adapted from McMichael (2001a), WHO (2002a).

**Table 2:** Examples of climate-sensitive exposures at all stages of development.

Preconception →	Embryo/fetus →	Newborn →	Juvenile →	Adolescence
Maternal nutritional status can affect lifetime risk of many chronic diseases (WHO 2002b).	Extreme heat during pregnancy is related to lower birth weight (Deschênes et al. 2009).	Breast-feeding practices are affected by extreme weather events (Akachi et al. 2009).	Diarrheal illness is already a leading cause of death in young children (Campbell-Lendrum and Woodruff 2006).	Particulate matter and ozone can affect lung development (Shea et al. 2007).

**Table 3:** Levels of prevention of climate change risks that have child-protective potential.

Prevention level	Goal	Child-protective examples
Zero-order	Prevent the development of a hazard	Urban planning to reduce GHGs from vehicular traffic and increase pedestrian access, resulting in improved air quality and increased physical activity (Sheffield and Galvez 2009)
Primary	Block interaction between hazard and human	Relocation of low-lying island populations facing increased risk of flooding, or distribution of mosquito nets
Secondary	Prevent effects after exposure to the hazard	Activation of early warning systems before heat waves
Tertiary	Reduce morbidity and mortality, avoid complications, and restore function	Postdisaster restoration of chronic care services

Adapted from Frumkin et al. (2008) and St Louis and Hess (2008).

**Table 4:** Children’s environmental health indicators (CEHIs) for major morbidity and mortality causes, selected for relation to climate change adaptation.

MEME model category	Perinatal diseases	Respiratory diseases	Diarrheal diseases	Physical injury	Insect-borne diseases
Contexts	------------------------------------ Children 0–14 years of age living in poverty ------------------------------------				Population growth rate in endemic disease areas

Exposures	Famine risk People living in informal settlements Malnourished women of childbearing age	Intrauterine growth retardation in newborns Children 0–14 years of age in unsafe housing	Drinking-water supplies failing national water quality	People living in informal settlements	Total area of insect vector habitats Children 0–14 years of age in households providing suitable conditions for insect-borne disease transmission
			------------ Children 0–14 years of age living in disaster-affected areas ------------		

Health outcomes	Intrauterine growth retardation in newborns	Morbidity rate for children 0–4 years of age due to acute respiratory illness	Diarrhea mortality and and morbidity in children 0–4 years of age	Mortality rate of children 0– 14 years of age due to physical illness	Prevalence of insect-borne diseases in children 0–14 years of age

Actions	Attributable change in number of households lacking basic services	Attributable change in number of households relying on biomass fuels or coal as the main source of heating and cooking	Attributable number of food outlets failing food hygiene standards	Children 0–14 years of age living within reach of specialist emergency medical services	At-risk children 0–14 years of age covered by effective, integrated vector control and management systems

MEME, multiple exposures and multiple effects. Adapted from WHO (2009a).
